# Validity of the LACE index for identifying frequent early readmissions after hospital discharge in children

**DOI:** 10.1007/s00431-021-03929-z

**Published:** 2021-01-15

**Authors:** Thang S Han, David Fluck, Christopher H Fry

**Affiliations:** 1 Institute of Cardiovascular Research, Royal Holloway, University of London, Egham, Surrey TW20 0EX UK; 2 Department of Endocrinology, Ashford and St Peter’s Hospitals NHS Foundation Trust, Guildford Road, Chertsey, Surrey KT16 0PZ UK; 3 Department of Cardiology, Ashford and St Peter’s Hospitals NHS Foundation Trust, Guildford Road, Chertsey, Surrey KT16 0PZ UK; 4 School of Physiology, Pharmacology and Neuroscience, University of Bristol, Bristol, BS8 1TD UK

**Keywords:** Healthcare services, Health economics, Quality improvement, LACE index

## Abstract

The LACE index scoring tool has been designed to predict hospital readmissions in adults. We aimed to evaluate the ability of the LACE index to identify children at risk of frequent readmissions. We analysed data from alive-discharge episodes (1 April 2017 to 31 March 2019) for 6546 males and 5875 females from birth to 18 years. The LACE index predicted frequent all-cause readmissions within 28 days of hospital discharge with high accuracy: the area under the curve = 86.9% (95% confidence interval = 84.3–89.5%, *p* < 0.001). Two-graph receiver operating characteristic curve analysis revealed the LACE index cutoff to be 4.3, where sensitivity equals specificity, to predict frequent readmissions. Compared with those with a LACE index score = 0–4 (event rates, 0.3%), those with a score > 4 (event rates, 3.7%) were at increased risk of frequent readmissions: age- and sex-adjusted odds ratio = 12.4 (95% confidence interval = 8.0–19.2, *p* < 0.001) and death within 30 days of discharge: OR = 5.0 (95% CI = 1.5–16.7). The ORs for frequent readmissions were between 6 and 14 for children of different age categories (neonate, infant, young child and adolescent), except for patients in the child category (6–12 years) where odds ratio was 2.8.

*Conclusion*: The LACE index can be used in healthcare services to identify children at risk of frequent readmissions. Focus should be directed at individuals with a LACE index score above 4 to help reduce risk of readmissions.

**Table:** 

**What is Known:** *• The LACE index scoring tool has been widely used to predict hospital readmissions in adults.*	
**What is New:** *• Compared with children with a LACE index score of 0–4 (event rates, 0.3%), those with a score > 4 are at increased risk of frequent readmissions by 14-fold.* *• The cutoff of a LACE index of 4 may be a useful level to identify children at increased risk of frequent readmissions.*

**What is Known:**

*• The LACE index scoring tool has been widely used to predict hospital readmissions in adults.*

**What is New:**

*• Compared with children with a LACE index score of 0–4 (event rates, 0.3%), those with a score > 4 are at increased risk of frequent readmissions by 14-fold.*

*• The cutoff of a LACE index of 4 may be a useful level to identify children at increased risk of frequent readmissions.*

## Introduction

It is well established that a small proportion of patients who are frequently readmitted to hospitals utilizes a large share of healthcare resources [1–3]. Moreover, the rate of emergency readmissions for UK children and young people in the 30 days following discharge from an emergency admission has been rising steadily, with an overall increase of 12% between 2006/2007 and 2015/2016. The increased readmission rate was observed in all age groups: 14% in those younger than 1 year old, 9% in 1–4-year-olds, 17% in 15–19-year-olds and 15% in 20–24-year-olds [4]. By contrast, over a similar period, the readmission rate after 30 days of discharge for US children aged 1–17 years was lower at 5.9%.

There are several models designed to predict the risk of paediatric readmissions. These include the All-Patient Refined Diagnosis-Related Groups [5] or a multivariate model for plastic surgery readmissions consisting of medical comorbidities, preoperatively contaminated or infected wound, higher American Society of Anesthesiologists scores, longer operative times and length of stay (LOS), and postoperative surgical and medical complications [6]. However, this model would not be applicable for our cohort as they contained several variables not necessarily measured in medical patients. The LACE index scoring tool is applicable to medical patients and is based on *L*ength of stay, *A*cuity of admission, *C*omorbidities and *E*mergency department visits, but was derived and is used for adults only to predict hospital readmissions and all-cause mortality within 30 days of hospital discharge [7]. However, there are no existing data on the ability of the LACE index to model readmissions in children. In particular, the cutoff score that predicts more frequent readmissions may be different for children compared to adults, because in a previous study we showed that the cutoff score in adults was dependent on patient age [8].

The objectives of this study were, in children less than 18 years of age, to (1) evaluate the ability of the LACE index to predict all-cause frequent early readmission rates (within 28 days of hospital discharge) and all-cause mortality; (2) derive a cutoff score for the LACE index, with the highest sensitivity and specificity, that predicts frequent readmissions; and (3) determine if the LACE index cutoff score for children is consistent with the age-dependent score measured in adults.

## Methods

### Study design, participants and setting

In line with the National Health Service (NHS), information on admission, discharge and readmission is recorded by Patient Administration System (PAS) which was accessed to identify all patients < 18 years old who were discharged alive between 1 April 2017 and 31 March 2019 from a single NHS hospital. A total of 6546 males and 5875 females from birth to 18 years were studied. Their clinical characteristics and care quality were recorded, including age, sex, primary diagnosis on admission, LOS in hospital, nature of the admission, comorbidities and the number of previous emergency department visits, as well as date of death of all-cause mortality after discharge from hospital.

### Measurement

Information on the frequency of unplanned readmissions within 28 days was recorded. Unplanned readmissions to hospital refer to those which are not planned or not from a waiting list. Comorbidities were coded according to the International Classification of Diseases (ICD-9) for calculation of a Charlson comorbidity index [9, 10]. Admissions for cancer and obstetrics were excluded in line with the NHS data collection system for general hospital admissions [11]. The LACE index was calculated from *L*ength of stay (score range 0–7), *A*cuity of admission (score 0 or 3), *C*omorbidities calculated by a Charlson comorbidity score [12, 13] (score range 0–5) with a lookback period within 12 months and *E*mergency department visits in the last 6 months (score 0 or 4), with an overall scale ranging from 0 to 19; the likelihood of outcome risk (early readmissions) is raised with increasing score [7, 14].

### Categorization of variables

Five age categories were created based on World Health Organization paediatric age categories: neonate (0–1 month), infant (1 month–2 years), young child (2–6 years), child (6–12 years) and adolescent (12–18 years) [15]. Readmissions within 28 days of discharge were categorized into three groups: no readmission, readmitted once and frequent readmissions (readmitted ≥ 2 times).

### Statistical analysis

Receiver operating characteristic (ROC) curves were constructed to determine the area under the curve (AUC) for the LACE index, as a predictor of all-cause frequent readmissions. Positive (LR+) and negative (LR−) likelihood ratios were calculated as $\frac{\mathrm{sensitivity}}{1\hbox{--} \mathrm{specificity}}$ and $\frac{1\hbox{--} \mathrm{sensitivity}}{\mathrm{specificity}}$, respectively. The minimum value of LR+ and the maximum value of LR− are 1 (unity). The further these values are from unity, the stronger the evidence for the presence or absence of poor outcome (readmissions), respectively. Two-graph ROC curve analysis was conducted to optimize the selection of the maximum test accuracy for a given LACE index threshold value to identify at-risk individuals—by plotting overlapping graphs of sensitivity and specificity curves as a function of LACE index scores. The threshold *d*_0_ was obtained by interpolating from the intersection where sensitivity equals specificity (*θ*_0_) [16–18], and also from the highest value of the average of sensitivity and specificity [17]. Precision-recall curves were also plotted to summarize the trade-off between the true positive rate and the positive predictive value. Precision was calculated as true positives/(true positives + false positives), and recall was the same as sensitivity. Residual analysis was used to assess the strength of the difference between observed and predicted numbers of patients with frequent readmissions at different LACE index scores using regression analysis [19, 20]. “Dummy variables” were created at two levels: 0 for ≤ 1 readmission (reference group), and 1 for ≥ 2 readmissions, and 0 for LACE index score = 0–4 (reference group) and 1 for LACE index > 4. Binary logistic regression was conducted to predict the risk of frequent readmissions or all-cause mortality within 30 days of discharge (the dependent variable) by LACE index score above the cutoff value derived from the two-graph ROC plot technique (the predictor variable). Multivariable Cox regression was used to predict mortality over a 2-year period and Kaplan-Meier curves were constructed to examine survival time after hospital discharge in relation to the LACE index. Analyses were performed first for all patients and then for different age categories, using IBM SPSS Statistics, v25.0 (IBM Corp., Armonk, NY).

## Results

The neonate group contained the highest proportion of patients (58.5%) and the child group the lowest (6–12 years, 8.4%), while other age groups comprised between 10 and 12% of patients. The proportions of overall patients with LACE index scores of 0–4, 5–9 and ≥ 10 were 82.0%, 17.8% and 0.2%, respectively. Most patients (93.8%) were not readmitted, with 5.4% readmitted once and only 0.9% readmitted ≥ 2 times within 28 days of hospital discharge. Except for neonates whose mean and median LACE index scores were 2, the corresponding scores were about 4 for infants, young children, children and adolescents, with a range of 0–10 (Table 1).

**Table 1:** Subject characteristics of 12,421 children (6546 males and 5875 females)

	*n*	%
Age distribution		
Neonates (0–1 month)	7271	58.5
Infants (1 month–2 years)	1466	11.8
Young children (2–6 years)	1380	11.1
Children (6–12 years)	1041	8.4
Adolescents (12–18 years)	1263	10.2
Length of stay in hospital		
0 day	4758	33.8
1 day	3735	30.1
≥ 2 days	3928	31.6
LACE index categories		
LACE index scores = 0–4	10,183	82.0
LACE index scores = 5–9	2212	17.8
LACE index scores ≥ 10	26	0.2
Number of readmissions within 28 days of discharge		
No readmission	11,645	93.8
Readmitted once	666	5.4
Readmitted ≥ 2 times	110	0.9
LACE index scores by age categories	Mean (median)	Range
Neonates (0–1 month)	2 (2)	0–10
Infants (1 month–2 years)	4.4 (4)	1–10
Young children (2–6 years)	4.0 (4)	0–10
Children (6–12 years)	4.2 (4)	0–10
Adolescents (12–18 years)	4.2 (4)	0–11

**Fig. 1:**
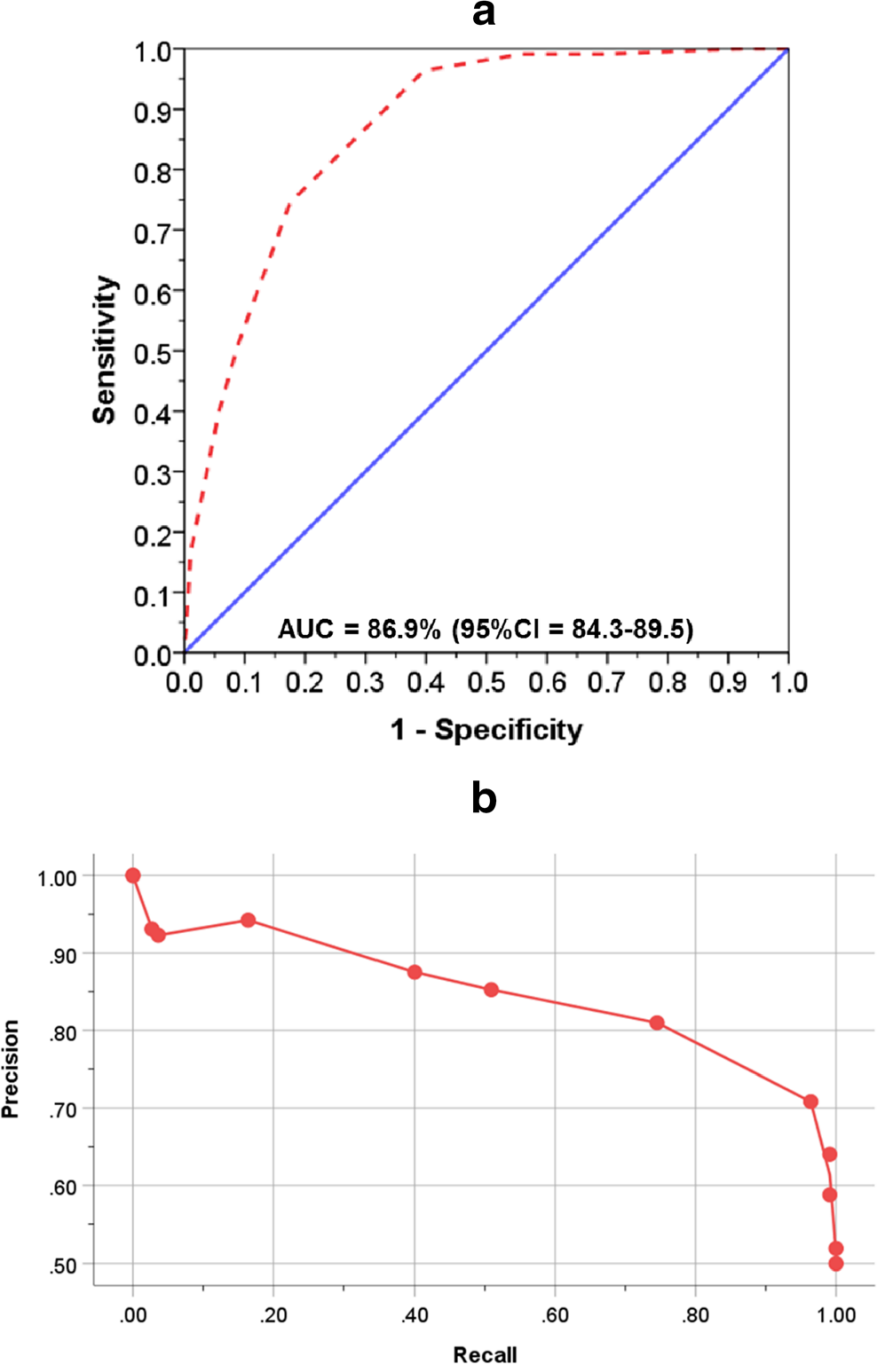
Receiver operating characteristic (**a**) and precision-recall (**b**) curves to estimate the ability of LACE index in the prediction of frequent readmission within 28 days after discharge from hospital in children

**Fig. 2:**
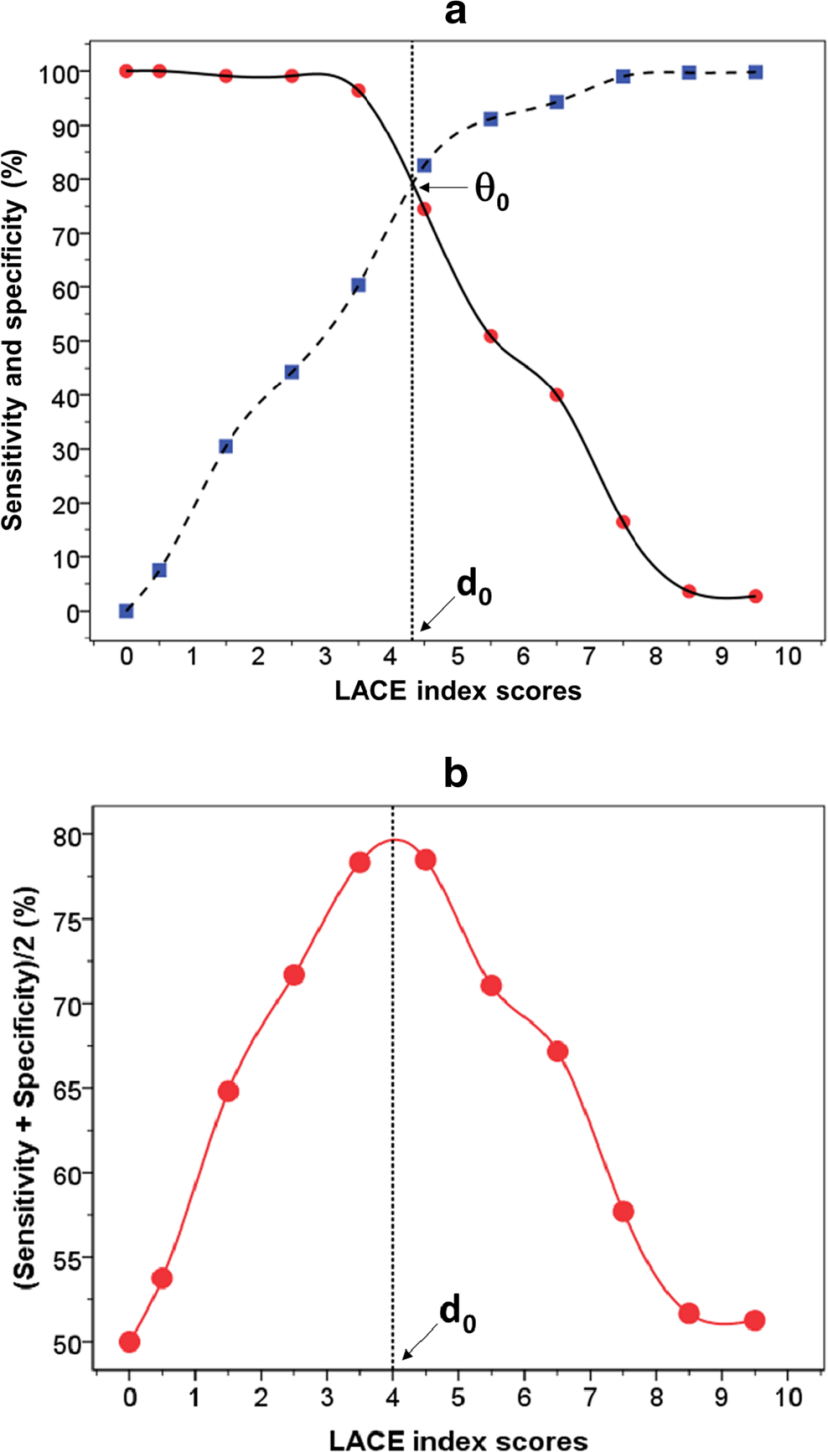
Two-graph ROC plot to identify frequent readmissions showing the threshold of LACE index (*d*_0_) interpolated from the point where sensitivity (●) equals specificity (■) (*θ*_0_) (**a**) and from the highest point of the average of sensitivity and specificity (**b**)

ROC curve analysis showed that the LACE index predicted frequent readmissions with a high degree of accuracy: the AUC was 86.9% (95% CI: 84.3–89.5%, *p* < 0.001) (Fig. 1a). The precision-recall curve shows the characteristic relationship between precision and recall (sensitivity): precision was about 80% at a recall level of 80; the precision-recall AUC was 84.3% (Fig. 1b). The LACE index threshold (*d*_0_) to predict frequent readmissions was interpolated from the intersection where sensitivity equals specificity (*θ*_0_) and was 4.3 (Fig. 2a). Similarly, a LACE index threshold of approximately 4.0 was identified by interpolating from the peak value of the average of sensitivity and specificity (Fig. 2b). A LACE index cutoff of 4 was applied to subsequent analysis to assess the risk of frequent readmissions: a LACE index score of 0–4 was considered a low risk (reference group) and > 4 as a high risk of frequent readmissions.

**Fig. 3:**
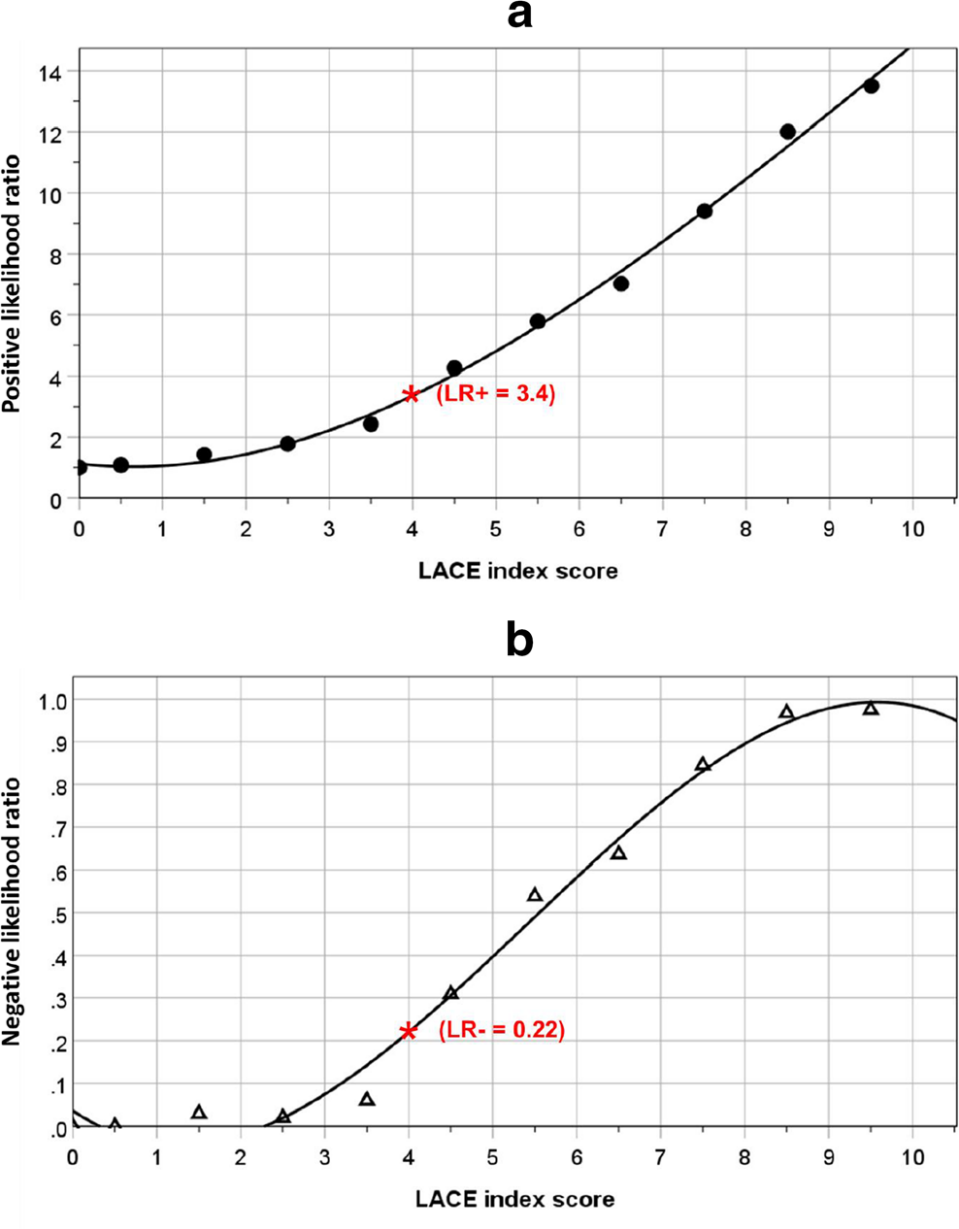
Relationship between LACE index scores and positive (**a**) and negative (**b**) likelihood ratios

Figure 3 shows curvilinear relationships between likelihood ratios and LACE index scores. Analysis using curve estimation revealed that a cubic model was the best fit, with LACE index scores explaining 99.7% of the total variance in LR+ and 98.9% in LR−. A sigmoid curve was more apparent for the LACE index and LR− relationship such that the LR− values were kept nearer to 0 until the LACE index score approached a value of 4, at which point the LR− values rose sharply towards unity. When the LACE index score was at 4, the LR+ was 3.4 and LR− was 0.22.

**Fig. 4:**
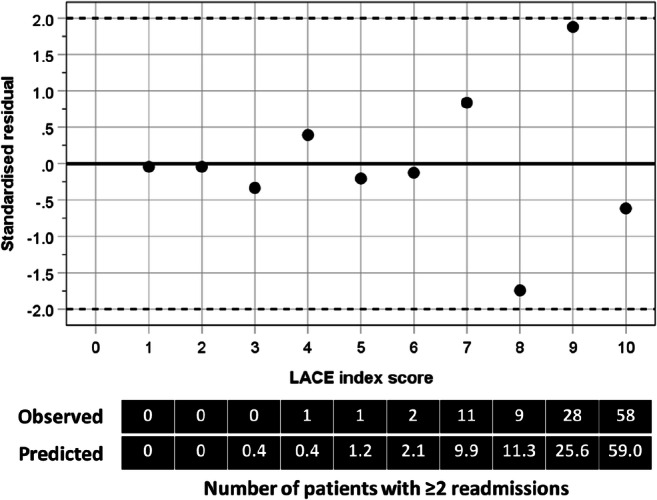
Plot of standardized residuals against LACE index scores

The observed numbers correlated highly with the predicted numbers of patients with frequent readmissions at different LACE index scores (*r* = 0.998, *p* < 0.001). Their standardized residuals were scattered randomly (*r* = 0.052, *p* = 0.883) around 0 (regression line) with no evidence for a trend in the spread of residuals with the LACE index scores. Most of the standardized residual values were close to 0 (within ± 1), except those at the LACE index scores of 8 and 9, but they were all distributed within the 95% limits of agreement (± 2) (Fig. 4).

Compared to patients with a LACE index score of 0–4, those with a score > 4 were at increased risk of frequent readmissions: age- and sex-adjusted odds ratio (OR) = 12.4, 95% confidence interval (95% CI) = 8.0–19.2 (*p* < 0.001). The ORs ranged between 6 and 14 for children of different categories (neonate, infant, young child and adolescent), except for those of child age (6–12 years) where the OR was 2.8; however, this group had only a small number of cases with frequent readmissions (*n* = 13) (Table 2).

**Table 2:** Logistic regression to assess the risk of frequent readmissions (≥ 2 times within 28 days after discharge from hospital, using one or less readmission within 28 days as reference group) among individuals with a LACE index score > 4

	Rates of frequent readmissions				Risk of frequent readmissions*					
	LACE index = 0–4 (reference group)		LACE index > 4		Unadjusted			Age- and sex-adjusted		
Unadjusted	*n*	%	*n*	%	OR	95% CI	*p*	OR	95% CI	*p*
All patients	28/10,193	0.3	82/2238	3.7	13.8	9.0–21.2	< 0.001	12.4	8.0–19.2	< 0.001
Age bands										
Neonates (0–1 month)	9/6607	0.1	13/664	2.0	14.6	6.2–34.4	< 0.001	5.7	2.2–15.2	< 0.001
Infants (1 month–2 years)	5/945	0.5	23/521	4.4	8.7	3.3–23.0	< 0.001	8.3	3.1–22.3	< 0.001
Young children (2–6 years)	4/1050	0.4	17/330	5.2	14.2	4.7–42.5	< 0.001	14.1	4.7–42.3	< 0.001
Children (6–12 years)	6/731	0.8	7/310	2.3	2.8^†^	0.9–8.4	0.067	2.8^†^	0.9–8.5	0.065
Adolescents (12–18 years)	4/850	0.5	22/413	5.3	11.9	4.1–34.8	< 0.001	11.7	4.0–34.2	< 0.001

*Reference group: LACE index score = 0–4. ^†^Males: OR = 12.2 (1.4–105, *p* = 0.023), females: OR = 1.0 (0.2–5.0, *p* = 0.952)

*OR*, odds ratio; *95% CI*, 95% confidence interval. “Dummy variables” were created at two levels: 0 for LACE index score = 0–4 and 1 for LACE index > 4, similarly, 0 for ≤ 1 readmission and 1 for ≥ 2 readmissions

Table 3 shows that among the top ten index diagnoses, the proportions of patients who were readmitted ≥ 2 times were significantly higher than those who were readmitted ≤ 1 occasion (*χ*^2^ = 43.7, *p* < 0.001). Inclusion of index diagnoses with fewer cases (between 50 and 60 cases) altered the results only slightly (*χ*^2^ = 50.0, *p* < 0.001).

**Table 3:** Proportions of patients presented with top ten index diagnoses who were readmitted ≤ 1 or ≥ 2 times within 28 days of discharge from hospital

Top ten diagnoses	*n*	Proportions of patients (%)	
		≤ 1 readmission	≥ 2 readmissions
Viral intestinal infection	64	0.5	0.9
Urinary tract infection	75	0.6	3.6
Constipation	80	0.6	0.9
Extremely low birthweight	80	0.6	0.9
Lobar pneumonia	121	1.0	0.9
Acute upper respiratory infection	122	1.0	1.8
Acute bronchiolitis	166	1.3	5.5
Asthma	167	1.3	3.6
Acute lower respiratory infection	235	1.9	4.5
Viral infection (unspecified)	701	5.6	6.4
Others	10,610	85.5	70.9
		Group differences: *χ*^2^ = 43.7, *p* < 0.001	

**Fig. 5:**
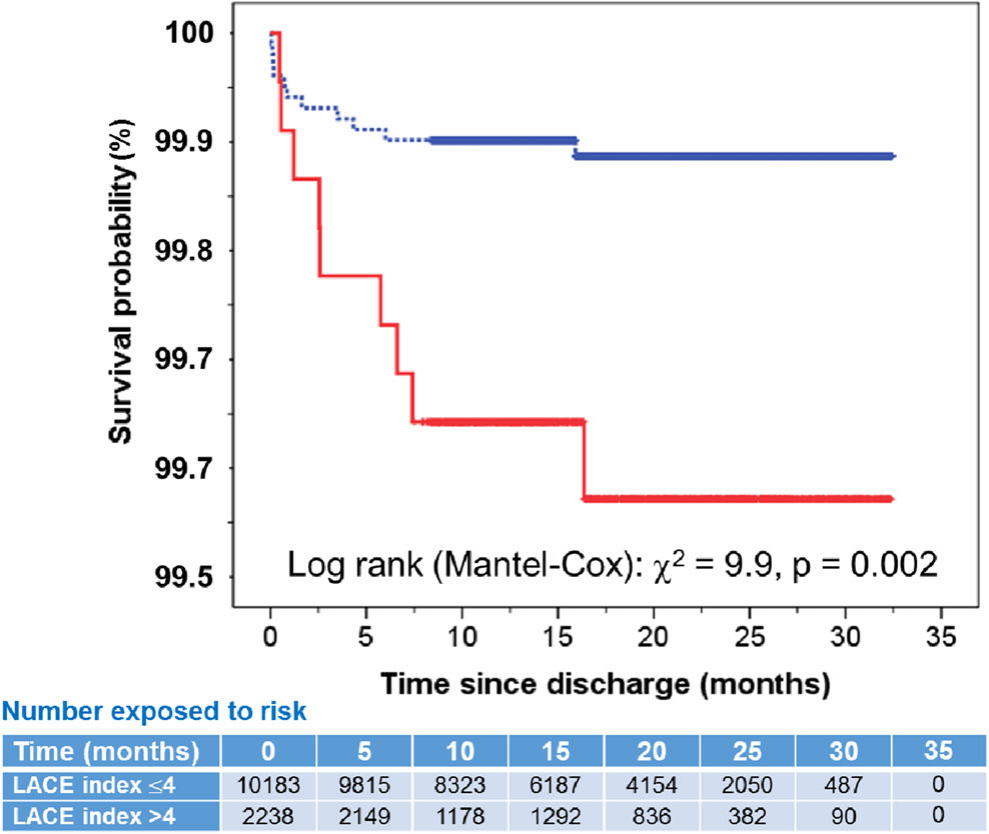
Kaplan-Meier survival plot in patients with LACE index scores = 0–4 (blue line) and > 4 (red line). The table beneath the figure shows the number of at-risk patients at various time points for the two LACE index cohorts

A total of 17 patients died within 30 days of discharge; the rate of death in patients with a LACE index score = 0–4 was 0.1% and those with a LACE index score > 4 was 0.3% (*χ*^2^ = 6.2, *p* = 0.022). Logistic regression showed that compared to patients with a LACE index = 0–4 (reference), those with a LACE index > 4 were at increased risk of death within 30 days of discharge: age- and sex-adjusted OR = 5.1 (95% CI = 1.5–16.7, *p* = 0.009). Over the period of study, there were proportionally higher rates of death among those with a LACE index score > 4 compared with those with a score = 0–4 (0.4% vs 0.1%), log rank (Mantel-Cox: *χ*^2^ = 9.9), age- and sex-adjusted hazard ratio = 4.09 (1.67–10.3, *p* = 0.003) (Fig. 5).

## Discussion

This study of children up to the age of 18 years showed that LACE index scores above 4 predict all-cause frequent early readmissions (≥ 2 times within 28 days after discharge from hospital). The risk of frequent readmissions was increased by 9- to 15-fold in the majority of children with a LACE index score above this value. As far as we are aware, this is the first study to demonstrate the predictive validity of the LACE index as a useful indicator to identify children at increased risk of frequent early readmissions.

The LACE index has been used widely in clinical practice across many countries, including the UK, to identify adult patients at risk of hospital readmissions. However, in adult patients, the LACE index performs this function with varying degrees of accuracy [21]. One reason for its effectiveness, or otherwise, will be the wide range of threshold score used in different studies, varying from 4 to 10 [21–24]. This may in part be driven by population demographics, as the cutoff score increases with age. There are few published studies on the association of the LACE index and readmissions in children. Ehwerhemuepha et al. [25, 26] showed that the LACE index performed modestly in the prediction of one or more readmissions within 30 days (AUC = 68–69.5%). However, we found a considerably higher AUC for LACE to predict frequent readmission that may in part be due to study criteria (readmissions were ≥ 2 in our study and ≥ 1 in those of Ehwerhemuepha et al. [25, 26]) and the fact that NHS emergency care is free to all, while health insurance is required in the USA. Thus, our findings may not be comparable to these other studies.

We recognize that the comorbidity component of the LACE index has less weight in children than in adults, so that LOS has a more dominant influence on the score than in adults. However, the LACE index performs better than using LOS alone. A ROC analysis for LOS alone showed an AUC of 75.9% (95% CI = 71.6–80.3) to predict frequent readmissions within 28 days. This should be compared to a value of 86.9% (95% CI = 84.3–89.5) for the LACE index and supports its use with children. We have also determined age-specific thresholds in children for the LACE index where sensitivity equals specificity, with values of 4, 5, 5, 4 and 5 for neonate, infant, young child, child and adolescent age groups, respectively, which all fall near to the threshold of 4 set in our study. The median LACE index scores were lowest in neonates and suggest a lower risk of readmissions compared with older children. Stratification by age groups showed the strength of the association between LACE index and frequent readmissions in neonates was similar to other age groups.

It is likely that children with complex conditions are more likely to be readmitted and more frequently [27]. In this study, we found that children who presented with the top ten index diagnoses including extremely low birthweight, constipation, asthma and childhood infections such as upper and lower respiratory infection and viral infections were proportionally higher among the frequent readmissions group compared with those who were readmitted once or less. Identifying patients at high risk of frequent emergency readmissions is crucial for healthcare planning. This would reduce preventable readmissions by ensuring safe discharge and implementing care support for patients in the community by specialist teams [28]. Over the period of study, there were proportionally higher rates of death among those with a LACE index score > 4 compared to those with a score = 0–4, and this suggests that an underestimation of the risk of frequent readmissions by the LACE index is likely.

**Fig. 6:**
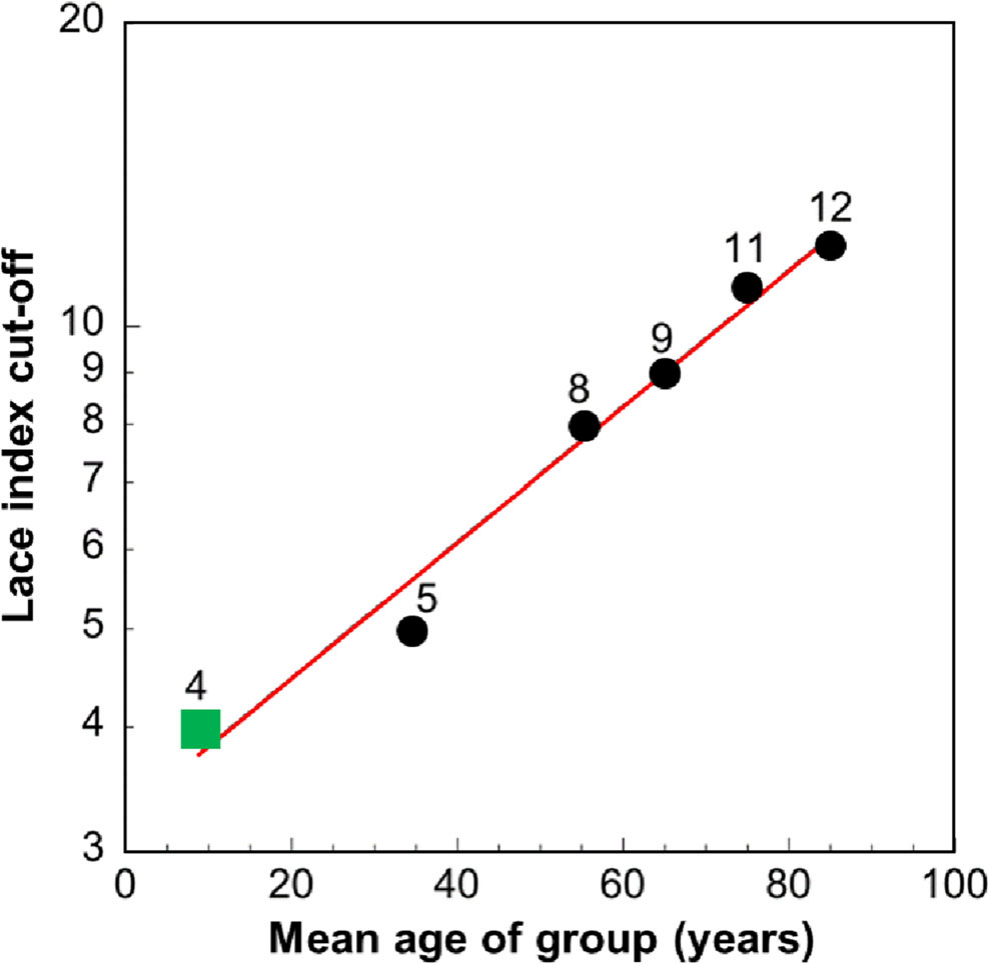
Relationship between LACE index cutoffs to predict frequent readmissions with age (semilog graph) in children (square) and adults (circles) of different age categories (data based on our study of adults [8])

No comparable study has been done previously with children and adolescents (aged < 18 years), and this study found that a value of above 4 provided a very high degree of accuracy in the prediction of early readmissions (AUC = 86.5%). This value also aligns with reducing the LACE index cutoff in younger adult groups: for example in the 18–50-year-old group, a value of 5 was determined. Figure 6 shows the LACE index cutoffs in children derived from this study, incorporated with those derived in adults from our other studies [8], and demonstrates the influence of age on LACE index cutoffs to predict frequent readmissions. Thus, there is a continuous relationship between the LACE index cutoff score and age that extends from the oldest adults to children. Unlike adults, we found that only 0.2% of children had a LACE index score of ≥ 10, whereas in adults ≥ 70 years, this value forms the cutoff level. A two-graph ROC plot technique to optimize the selection of the maximum test accuracy also revealed a LACE index threshold to be at 4 in the identification of at-risk individuals. This level was again corroborated by a LACE index threshold obtained from the highest average value of sensitivity and specificity.

A study of 44,500 children aged 0–16 years admitted to two London district hospitals between 2009 and 2010 showed between 3 and 4% presented ≥ 4 times during a 1-year period [29]. However, there is in general a paucity of data on frequent early hospital readmissions for children. We found relatively small percentages of readmissions among children in our study, with 5.4% readmitted once and 0.9% readmitted ≥ 2 times within 28 days after a hospital discharge. Thus, efforts to prevent frequent early readmissions in this small group of high-risk individuals would be justifiable in order to reduce morbidity in them and ameliorate pressure on healthcare services.

A recent estimate of admission and readmissions for US children was $1.58 billion, of which about 43% of these costs were attributable to readmissions [30]. The average costs of readmission stay in this study was $13,400, but varied from $5000 for asthma to $39,500 for septicaemia [30]. The ability to identify such patients is crucial to allow effective clinical management to be planned and to reduce frequent readmissions.

Likelihood ratios are useful statistics for measuring the accuracy of a diagnostic test in a clinical setting [31]. The likelihood ratio is derived from the ratio of the probability of a given test result in patients with poor outcome (i.e. frequent readmissions) to the probability of the same test result in patients with a good outcome. Generally, an LR+ value greater than 10 and LR− value lower than 0.1 indicate strong evidence to confirm or exclude the diagnosis of interest, respectively [32]. The random spread of standardized residuals around the regression line indicates good agreement between the observed and predicted values of readmissions, but at higher LACE scores (above 7) the observed deviated more from the expected and therefore calibrates less for children with higher scores. However, the chosen LACE index cutoff of 4 lies well below that range. This further supports the validity of applying the LACE index to children. We recognize that the LACE index scores are continuous measurements but their relationships with likelihood ratios are curvilinear, such that there was an inflexion point for LR− which appeared at the LACE index score of 4, supporting the observations from the two-graph ROC analysis.

The selection of a LACE index cutoff for clinical practice should be based on the balance of available resources and risk to patients. Varying the LACE index threshold would change the sensitivity (recall), specificity and precision of prediction, as well as LR+ and LR− values. If the LACE index threshold were raised above 4, then total numbers would be smaller, with a greater proportional readmission rate, i.e. there would be fewer false negatives, but precision will diminish. On the other hand, by staying at 4 or below, more false positives are introduced and some true positives will be missed, but precision will be high.

The strengths of this study lie in its large number of consecutive patients, which enable us to estimate the risk of mortality by age categories, ranging from birth to 18 years. There are certain limitations in the present study. This is single-centred study; therefore, it did not capture readmissions to other hospitals which may have led to an underestimation of readmissions. We used 28 days to define the period for emergency readmissions as guided by the NHS [3], while some studies have used 30 days for their definition [33], which would capture slightly higher numbers of readmissions. However, these differences do not affect the outcome of our studies since the primary purpose of this study was not intended for comparison of the performance of the LACE index with other indicators. We did not include patients with cancers which may have some bearing on our results. It is likely that if cancer cases were included, the predictive power of the LACE index would increase further, since children with cancer have been shown to be readmitted frequently within 1 year after their first admission [34]. We set the criteria for frequent readmissions as ≥ 2, since 1 readmission is not considered as frequent. There would be more patients in the risk category if ≥ 1 readmission was used, but this would diminish the association with the LACE index. Although the LACE index was originally derived from the adult population, our study has shown its potential as a predictor of frequent readmissions in children. However, before the LACE index could be confidently applied in clinical practice, further studies are necessary in an independent children population to cross-validate our findings.

In conclusion, the LACE index predicts frequent early readmissions with high accuracy, and the cutoff of LACE index of above 4 may be a useful level to identify children at increased risk of frequent readmissions.

## Data Availability

N/A.

## Abbreviations

AUC: Area under the curve
CI: Confidence interval
ICD: International Classification of Diseases
LACE: *L*ength of stay, *A*cuity of admission, *C*omorbidities and *E*mergency department visits
LOS: Length of stay
LR: Likelihood ratio
NHS: National Health Service
OR: Odds ratio
ROC: Receiver operating characteristic
